# Medication adherence, medical record accuracy, and medication exposure in real-world patients using comprehensive medication monitoring

**DOI:** 10.1371/journal.pone.0185471

**Published:** 2017-09-28

**Authors:** Timothy P. Ryan, Ryan D. Morrison, Jeffrey J. Sutherland, Stephen B. Milne, Kendall A. Ryan, J. Scott Daniels, Anita Misra-Hebert, J. Kevin Hicks, Eric Vogan, Kathryn Teng, Thomas M. Daly

**Affiliations:** 1 Sano Laboratories, Sano Informed Prescribing, Franklin, Tennessee, United States of America; 2 Department of Internal Medicine, Cleveland Clinic, Cleveland, Ohio, United States of America; 3 Medicines Department, Cleveland Clinic, Cleveland, Ohio, United States of America; 4 Reporting and Analytics, Cleveland Clinic, Cleveland, Ohio, United States of America; 5 Medicines Department, Cleveland Clinic, Cleveland, Ohio, United States of America; 6 Robert J. Tomsich Pathology and Laboratory Medicine Institute, Cleveland Clinic, Cleveland, Ohio, United States of America; Universite de Bretagne Occidentale, FRANCE

## Abstract

**Background:**

Poor adherence to medication regimens and medical record inconsistencies result in incomplete knowledge of medication therapy in polypharmacy patients. By quantitatively identifying medications in the blood of patients and reconciling detected medications with the medical record, we have defined the severity of this knowledge gap and created a path toward optimizing medication therapy.

**Methods and findings:**

We validated a liquid chromatography-tandem mass spectrometry assay to detect and/or quantify 38 medications across a broad range of chronic diseases to obtain a comprehensive survey of patient adherence, medical record accuracy, and exposure variability in two patient populations. In a retrospectively tested 821-patient cohort representing U.S. adults, we found that 46% of medications assessed were detected in patients as prescribed in the medical record. Of the remaining medications, 23% were detected, but not listed in the medical record while 30% were prescribed to patients, but not detected in blood. To determine how often each detected medication fell within literature-derived reference ranges when taken as prescribed, we prospectively enrolled a cohort of 151 treatment-regimen adherent patients. In this cohort, we found that 53% of medications that were taken as prescribed, as determined using patient self-reporting, were not within the blood reference range. Of the medications not in range, 83% were below and 17% above the lower and upper range limits, respectively. Only 32% of out-of-range medications could be attributed to short oral half-lives, leaving extensive exposure variability to result from patient behavior, undefined drug interactions, genetics, and other characteristics that can affect medication exposure.

**Conclusions:**

This is the first study to assess compliance, medical record accuracy, and exposure as determinants of real-world treatment and response. Variation in medication detection and exposure is greater than previously demonstrated, illustrating the scope of current therapy issues and opening avenues that warrant further investigation to optimize medication therapy.

## Introduction

The United States spends more on healthcare and prescribes more medications per patient than any other country [1, 2]. Despite this, health outcomes in the United States are poor compared to other industrialized countries. The greatest portion of expenditure is for chronic conditions; for example, in 2013 diabetes ranked first in overall healthcare spending at over $100 Billion, and of that cost, more than 57% was driven by pharmaceuticals [3]. Although diabetes medications have proven to be efficacious in clinical studies, the effectiveness of these and other medications must be improved, as there is a disconnect between drug efficacy in controlled clinical trials and effectiveness in real-world patient settings [4]. Lack of medication effectiveness may result from poor patient behavior, healthcare delivery flaws, inter-individual variability in medication response, or a combination of these factors [5, 6]. To better understand medication effectiveness, it is vital to know if patients are compliant with prescribed medication regimens, if the medical record used by the healthcare provider is accurate, and if medication concentrations are within target blood ranges. Knowing the medication concentration in blood is particularly relevant to medication effectiveness and has demonstrated treatment utility, particularly in the field of psychiatry [7]. Levels below the therapeutic reference range may not provide therapeutic benefit, while levels above the therapeutic reference range may increase the risk of adverse events without offering additional benefit.

While adherence to test medications in clinical trials is typically high, the post-FDA-approval reality is that real-world patient adherence is variable and difficult to measure [8, 9]. Adherence to medication treatment regimens is driven by economic, health literacy, side effect profiles, or a host of other factors [10, 11]. Approximately 25% of patients do not pick up their medications after the initial prescription, and 40% do not refill prescriptions for medications prescribed for chronic conditions. The cost to the healthcare system of nonadherence is staggering, estimated to be greater than $200 billion, largely driven by avoidable hospitalizations [12]. A recent study by Kymes et. al., demonstrated the benefit of addressing patient adherence, showing cost savings in the thousands of dollars annually for co-morbid patients when adherence was improved. Moreover, this study and others have demonstrated that persistence—keeping adherent patients adherent—was largely responsible for the savings incurred [13–16].

The electronic monitoring of medication container usage may represent the gold standard for assessing medication adherence, surpassed only by direct observation of medication intake [17]. Objective direct methods, such as unscheduled blood monitoring, may be attractive, but these methods have been mostly limited to testing for drugs of abuse. Furthermore, there are documented studies of improved adherence shortly before physician appointments, demonstrating the need to measure adherence in real-world workflows to determine the impact on hospitalizations, ED visits, and other outcomes. Indeed, improving how current medicines are taken could have far reaching implications on outcomes; maybe more so than newly developed treatments [18].

Each patient’s accounting of medications is located within their electronic health record (EHR). Complex patients often have multiple healthcare professionals using separate EHR systems, each of which provide an incomplete view of the patient’s care. Using patient pharmacy records, the EHR, and patient interviews, discrepancies were observed in over 33% of patients when assessed at hospital admission [19]. When reconciliation was led by a trained pharmacist, post-hospitalization healthcare utilization was improved, including hospital revisits, emergency department visits and hospital readmissions [20, 21]. When delivered as an integrated solution, adherence intervention and medical record reconciliation represent opportunities for innovation that can un-blind the healthcare provider to the patient’s true treatment regimen.

Therapeutic drug monitoring has been an effective means to improve therapy for select medications, typically those with narrow therapeutic margins. When coupled with genetics, therapeutic drug monitoring can identify causes as to why medications do not fall within therapeutic reference ranges, and can be used to guide medication selection or dosage changes [22–25]. A properly attained circulating exposure measurement offers a surrogate biomarker of drug action and can minimize the guess-work often associated with dose selection [26]. The measurement of medication concentrations takes into consideration all sources that impact exposure, as these measurements are the manifestation of variability in patient treatment and response. Historically, therapeutic drug monitoring has been impractical for polypharmacy patients due to cost, pharmacokinetic considerations, and sample volume necessary to cover the wide spectrum of medicines. In addition, current approaches to therapeutic drug monitoring are limited in their scope and can be criticized as “looking under the streetlight”, missing medications that are unknown to the physician. Improvements in medication monitoring technology using sensitive, high-throughput approaches [27, 28] have now made it possible to comprehensively assess multiple medications simultaneously and assess total medication burden in the polypharmacy patient.

Herein we utilized a liquid chromatography-tandem mass spectrometry (LC/MS/MS) assay capable of quantifying 38 medications from multiple medication classes in a single blood sample. We assessed medication exposure at the time of sample collection, and subsequently matched the detected medications with the primary medical record. We quantified medications in two distinct patient cohorts, each to answer a different question. First, by performing the comprehensive medication test during visits to healthcare facilities where medication testing was not anticipated, we explored the use of medication detection as an unambiguous measure of real-world adherence to ascertain the fidelity of the medical record. In a second cohort, we measured medication concentrations in prospectively enrolled, adherent patients with reconciled medical records, comparing the measured concentration of each detected medication to established reference ranges. By enrolling adherent patients and reconciling records prior to testing, we were able to explore exposure variability for the 38 drugs queried. The present investigation is the first to empirically assess compliance, medical record accuracy, and exposure as determinants of real-world treatment and response in complex patients, providing insight to the scope of current therapy issues and potential avenues to optimize medication therapy.

## Materials and methods

### Clinical samples

The two studies included in this report were conducted at the Cleveland Clinic, Cleveland OH. Trials were conducted by Cleveland Clinic personnel and approved by the Cleveland Clinic Institutional Review Board. All patients provided written informed consent, and for patients below the age of 18, informed consent was obtained by parent or legal guardian. Patient enrollment began in April 2015 and last patient visit was in September 2015. All samples were collected from both cohorts within this timeframe. Sample analysis was performed by Sano Informed Prescribing Inc., Franklin, TN.

A patient cohort representative of U.S. hospital patients (Residuals Cohort) was obtained by randomly selecting residual samples from patients receiving a Vitamin D test. Vitamin D testing was chosen because it is a high-volume test that is routinely ordered in otherwise generally healthy outpatients. The Cleveland Clinic central electronic health record database was utilized to match medication lists with residual serum samples from 1000 subjects. Samples with non-unique identifiers or origin numbers that did not match extraction criteria were excluded from the analysis. The resulting cohort consisted of 821 patients with available serum and a matching medication list. A second patient cohort (Reconciled Cohort) with improved adherence and demonstrated polypharmacy was obtained by prescreening medication lists from patients prescribed at least five overall medications, including at least two medicines represented in the test panel and one medicine of the psychotropic drug class. These enrollment criteria, coupled with an interview-based reconciliation of the medical record prior to admission, blood draw, and analysis created a biased cohort with improved medication adherence and demonstrated polypharmacy. Adherence improvement was likely a result of: 1) removing medications within the EHR no longer taken by the patient based on interview and 2) consent bias toward more adherent patients. Approximately 500 patients were approached based on pre-enrollment criteria resulting in a final cohort of 151 patients.

For both study cohorts, serum samples were transferred into microsample tubes bearing study-specific identifiers. The key linking study-specific identifiers to EHR information was maintained by study personnel at the Cleveland Clinic and not shared externally. Serum samples were stored at -70°C, until shipping to Sano Informed Prescribing Laboratories for LC/MS/MS analysis. The medications measured in the assay were prescribed for the treatment of psychiatric disorders, idiopathic or anatomical pain, cardiovascular disease, diabetes, and gastrointestinal complications. Sano Informed Prescribing, Inc. is accredited through the College of American Pathology (CAP# 9265097) and CLIA registered (44D2096427). Sample analysis was executed under the guidelines set forth by the CAP and standard operating procedures commensurate with CLIA-registered operations.

Sano laboratory personnel were blinded to study participants’ records and reported medications during the measurement phase of the studies. After measurement, deidentified medication lists from the EHRs were compared to LC/MS/MS measured results and classified into one of the following three categories: 1) detected and prescribed (DAP); 2) prescribed, but not detected (PND); or 3) detected, but not prescribed (DNP). Additional analyses included the comparison of quantitative measurements for each detected medication to serum reference ranges available in the literature (S1 Table).

### Reagents and standards

Optimal grade methanol and acetonitrile were obtained from Fisher Scientific (Waltham, MA). Formic acid, ammonium acetate, ammonium formate, and water were all LC/MS grade and obtained from Sigma-Aldrich (St. Louis, MO). Dimethylsulfoxide was obtained from Sigma-Aldrich. Ammonium hydroxide was obtained from Thermo Fisher Scientific. Drug naïve human serum used in validation studies was obtained from Bioreclamation IVT (Westbury, NY). All analytical standards were obtained at the highest purity available. Stock solutions were prepared individually in DMSO, water, methanol, or acetonitrile, then combined. Standard Curve and Quality Control samples were prepared in drug naïve human serum.

### Sample extraction

Serum samples were collected in red top gel barrier-free microsample tubes, frozen, and shipped on dry ice to Sano Informed Prescribing for processing. Samples were thawed, mixed, and transferred to 96-well plates for processing. Internal standard working solution was added and protein precipitation was performed using Phenomenex Impact Protein Precipitation Plates. Eluate was transferred to a new plate and dried under Nitrogen. Sample was reconstituted for LC/MS/MS analysis.

### LC/MS/MS analysis

Reconstituted samples were processed using a Shimadzu Nexera X2 liquid chromatography system (Columbia, MD)) fitted with a 2.1 x 50 mm, 1.7um C18 column (Phenomenex, Torrence, CA)). Sample analysis was performed on a Sciex 5500 QTrap Mass Spectrometer (Framingham, MA) with TurboV ion source and polarity switching. Data collection was performed with Sciex Analyst software, version 1.6.2, and data analysis was performed using Indigo BioAutomation Ascent software (Indianapolis, IN).

Assay linearity, precision, accuracy, and detection were validated by adding various amounts of each test drug to human serum. Each of the 38 drugs assayed passed strict analytical validation criteria. Three medications originally intended to be included in the multi-plex assay exhibited poor analytical performance and were excluded from analysis. Bupropion exhibited plasma instability, and lovastatin and phenytoin exhibited poor performance near the lower levels of the therapeutic reference range necessary for data interpretation. The final number of medications tested and included in all analyses was 38 (S1 Table).

### Quantitative medication reporting

Reference ranges for each of the 38 parent drugs were obtained using triaged data sources as indicated in S1 Table. The primary information source was obtained from the AGNP Consensus Guidelines for Therapeutic Drug Monitoring in Psychiatry, which is a comprehensive, evidence-based summary of therapeutic reference ranges for 128 marketed medications. If the medication was not listed in this primary source, secondary sources derived from primary literature were utilized. Finally, if no literature values could be obtained, drug label information was utilized [29–35]. Medications were mapped to drug classes according to the NHANES resource (https://wwwn.cdc.gov/nchs/nhanes/1999-2000/RXQ_DRUG.htm; accessed 3/9/2017.

## Results

We developed a multiplex assay for the quantitative assessment of serum concentrations for medications used clinically in the management of chronic disease. The 38-medication panel was biased toward medications that target the central nervous system, with the balance prescribed for cardiovascular, metabolic, or gastrointestinal indications. Over-the-counter and non-centrally acting medications were selected that are known to be co-prescribed at high rates with psychotropic medications [36], known perpetrators of drug interactions, or metabolized through pathways with documented genetic influence. Both acute-acting and chronic-acting medications were included in the test panel. The average coefficient of variation (CV) established for quality control was less than 20% for the lower (17.3%) and upper range (16.8%) of quality control samples. The therapeutic reference range, as defined in Hiemke et.al. [26], was determined for each medication from literature. Measures of inter-assay precision and accuracy for each analyte and corresponding range parameters are presented in S1 Table. Nearly all medications in the assay were detected in at least one patient, except for gemfibrozil, which was prescribed three times but never detected, and clozapine/phenytoin that were not prescribed or detected in either patient cohort.

Two patient cohorts were selected to answer separate questions pertaining to medication treatment and pharmacokinetic response. The first cohort consisted of 821 patients randomly selected from routine clinical testing for serum Vitamin D levels (Residuals Cohort). Patients ranged in age from 5 to 103 years, with an average age of 54. In 39% of patients, zero panel medications were detected and 4% of patients had five or more panel medications detected. A second cohort consisting of 151 patients with documented polypharmacy, including at least one psychotropic medication, was prospectively enrolled based upon prescreening criteria (Reconciled Cohort). Owing to the selection criteria, 19% of patients had five or more detected panel medications. Enrollment criteria for this cohort created a strong bias of 78% female patients with an average age of 57. Patient characteristics and summary medication results are listed in Table 1.

**Table 1:** Characteristics of patient cohorts.

	Residuals Cohort	Reconciled Cohort
**Demographics**		
Total Subjects	821	151
Male Subjects	34%	22%
Female Subjects	66%	78%
Average Subject Age	54	57
Youngest Subject	5	24
Oldest Subject	103	75
**Prescriptions and detections**		
Average prescribed medications in assay	1.5	3.4
Fewest prescribed medications in assay	0	1
Most prescribed medications in assay	7	7
Average detected medications in assay	1.3	3.2
Fewest detected medications in assay	0	0
Most detected medications in assay	8	8

**Fig 1:**
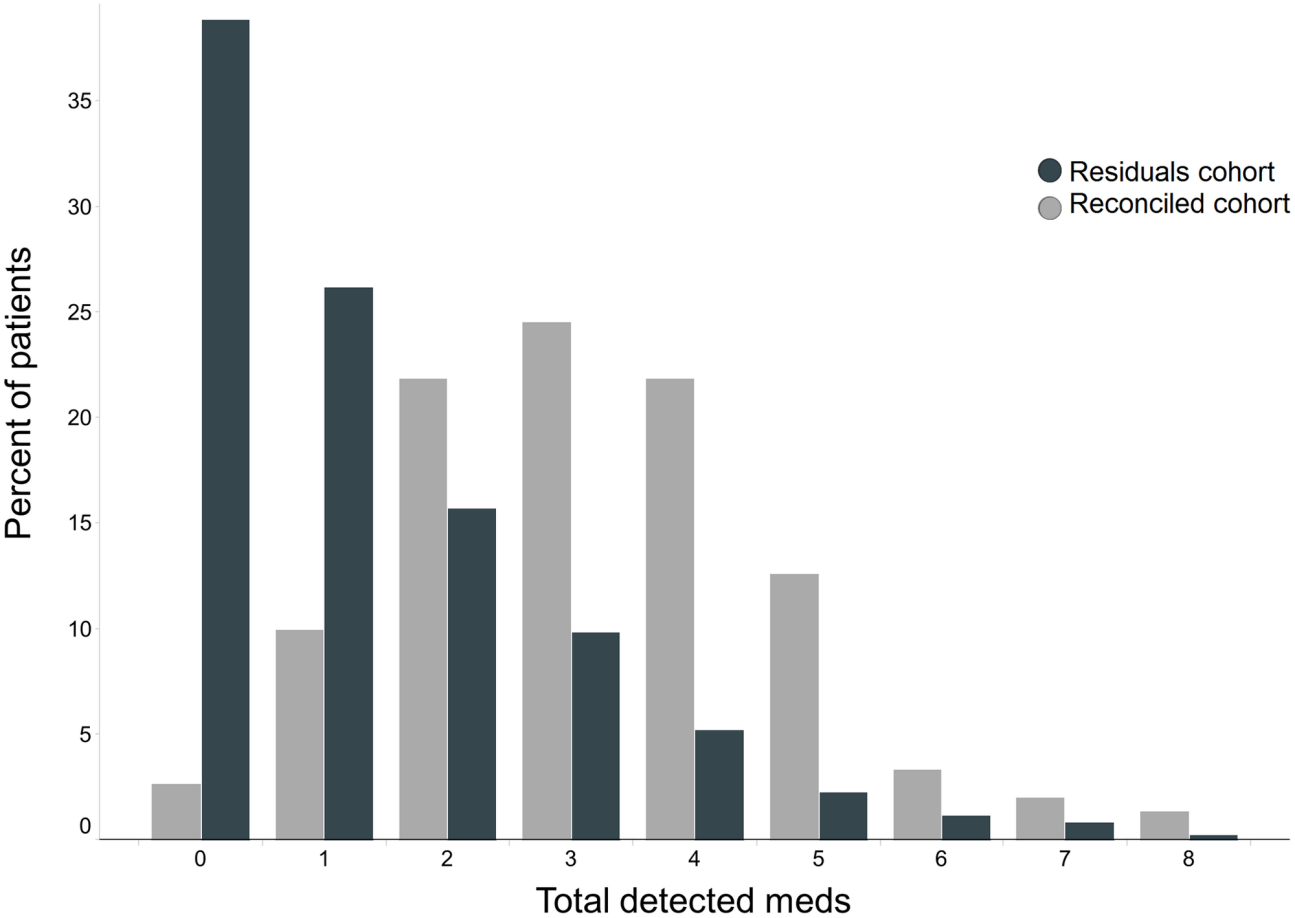
Distribution of total detected medications for two cohorts. Percent of patients having between 0 and 8 detected medications in the Residuals vs. Reconciled cohorts.

**Fig 2:**
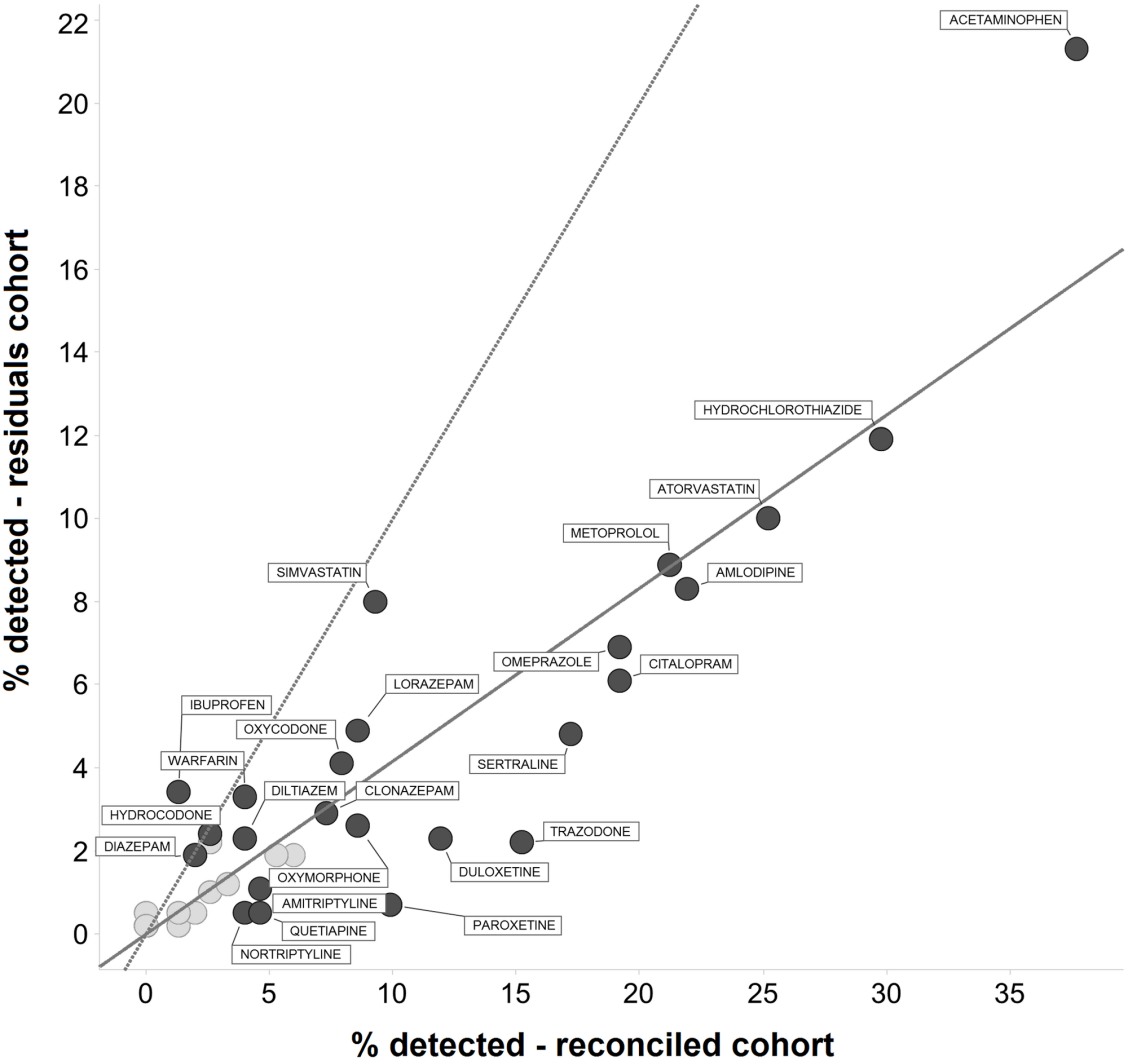
Detection rate for panel medications in two cohorts. Percent of patients for whom a given medication is detected in Residuals vs. Reconciled Cohorts. The dotted line indicates equal detection rates in both cohorts, while the solid line indicates the ratio of overall detection rate in both cohorts: 1.3 detected drugs per patient in Residuals Cohort vs. 3.2 detected drugs per patient in Reconciled Cohort.

The distribution of total number of detected medications differed significantly across the cohorts (p = 1e-14, Mann-Whitney U-test; Fig 1), with more medications detected per patient in the prospectively enrolled Reconciled Cohort. Across individual patients, the number of detected drugs was correlated with the number of prescribed drugs (Spearman ρ = 0.61 and 0.69 in Residuals and Reconciled cohorts, respectively; S1 Dataset). The rate of detection for individual drugs was correlated in the two cohorts (Spearman ρ = 0.81), although the median rate of detection in the Reconciled Cohort was 2.4 times greater (Fig 2). Psychotropic medicines were detected at an even greater rate in the Reconciled Cohort, which required at least one psychotropic medication for enrollment.

**Fig 3:**
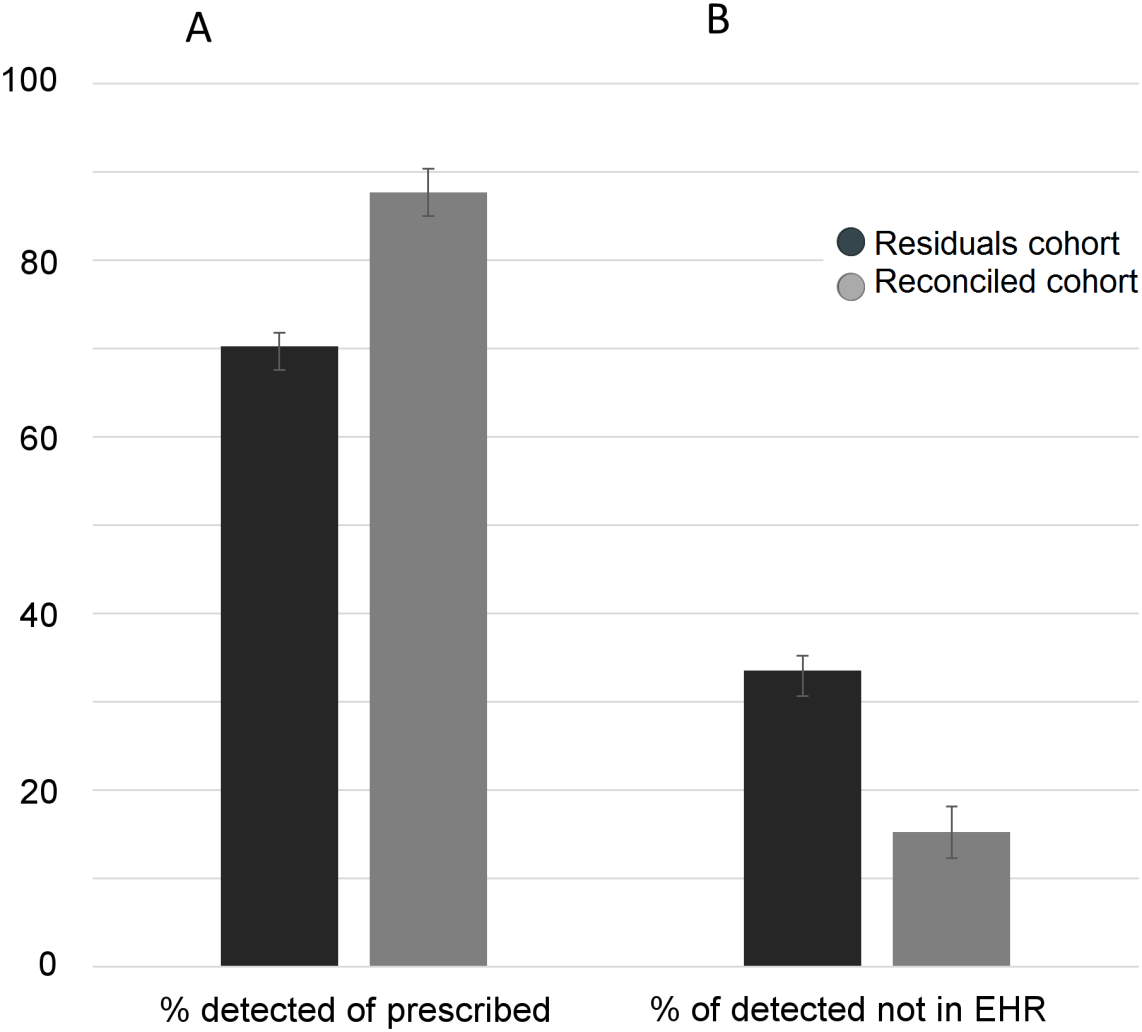
Medication prescriptions according to EHR vs. medication detection in two cohorts. A) Percent of prescribed medications that are detected and B) percent of detected medication that are non-prescribed (i.e. not in the EHR). Error bars were calculated from Bernoulli trials.

We tabulated drugs across categories denoting whether each detected medication was consistent with the medication list in the patient’s EHR (Table 2). There were three potential scenarios. A medication could be detected and prescribed (DAP), prescribed but not detected (PND), or detected but not prescribed (DNP). For drugs that were prescribed but not detected, we identified and removed the subset that were prescribed on an ‘as needed’ basis (PND prn), because failure to detect such medications could not be used as a surrogate measure of non-adherence. We noted that the proportion of prescribed medications that were detected was significantly higher (Fig 3A, p = 3e-13, two-sided χ^2^-test), and the proportion of detected medications not in the medical record was significantly lower (Fig 3B, p = 7e-14, two-sided χ^2^-test) in the Reconciled Cohort relative to the Residuals Cohort. These trends further illustrate bias from the Reconciled Cohort enrollment criteria. Within this Cohort the number of medications prescribed not detected was similar for males vs. females (93% vs. 86%, p = 0.06, two-sided χ^2^-test).

**Table 2:** Medication panel characteristics and detection relative to prescription record for two patient cohorts.

Medication	t1/2 (hr)	Residuals Cohort			Reconciled Cohort		
		DAP^a^	DNP^b^	PND^c^	DAP^a^	DNP^b^	PND^c^
		**Analgesics**					
acetaminophen	2	48	127	15	28	29	0
dihydrocodeine	3.5	0	2	0	0	0	0
hydrocodone	4	10	10	5	4	0	0
hydromorphone	2.4	3	1	0	0	2	0
ibuprofen	2	8	20	7	1	1	0
oxycodone	3.5	22	12	1	10	2	3
oxymorphone	NA	0	21	1	0	13	1
		**Antidepressants**					
amitriptyline	19	9	0	7	7	0	1
citalopram	33	41	9	8	24	5	2
duloxetine	14	18	1	9	17	1	4
fluoxetine	120	13	3	2	8	1	0
nortriptyline	30	3	1	2	5	1	1
paroxetine	28	6	0	6	13	2	2
sertraline	23	33	6	8	25	1	2
trazodone	7.5	12	6	10	23	0	1
		**Antipsychotics**					
clozapine	14	0	0	0	0	0	0
olanzapine	45	1	3	0	3	0	0
quetiapine	7	4	0	3	7	0	2
		**Benzodiazepines**					
clonazepam	40	15	9	3	11	0	0
diazepam	36	9	7	1	2	1	0
lorazepam	14	15	25	4	11	2	0
alprazolam	13.5	10	6	5	8	0	0
oxazepam	9.5	0	2	0	0	0	0
temazepam	9	1	1	1	1	1	0
		**Cardiovascular/Metabolic**					
amiodarone	75	2	2	1	0	0	0
amlodipine	42	63	5	21	31	2	2
atorvastatin	19.5	69	13	28	37	1	3
clopidogrel	2.5	7	3	11	5	0	1
diltiazem	4	14	5	1	5	1	0
gemfibrozil	1.1	0	0	2	0	0	1
hydrochlorothiazide	11	90	8	27	45	0	2
metoprolol	5	61	12	15	31	1	1
pravastatin	2.9	15	3	16	4	0	6
simvastatin	2.5	40	26	27	12	2	10
verapamil	4	5	3	2	4	0	1
warfarin	43.5	26	1	4	5	1	1
		**Other**					
omeprazole	1	45	12	50	25	4	11
phenytoin	40	0	0	0	0	0	0

Abbreviations used

^a^ detected and prescribed (DAP),

^b^ detected not prescribed (DNP),

^c^ prescribed not detected (PND)

**Fig 4:**
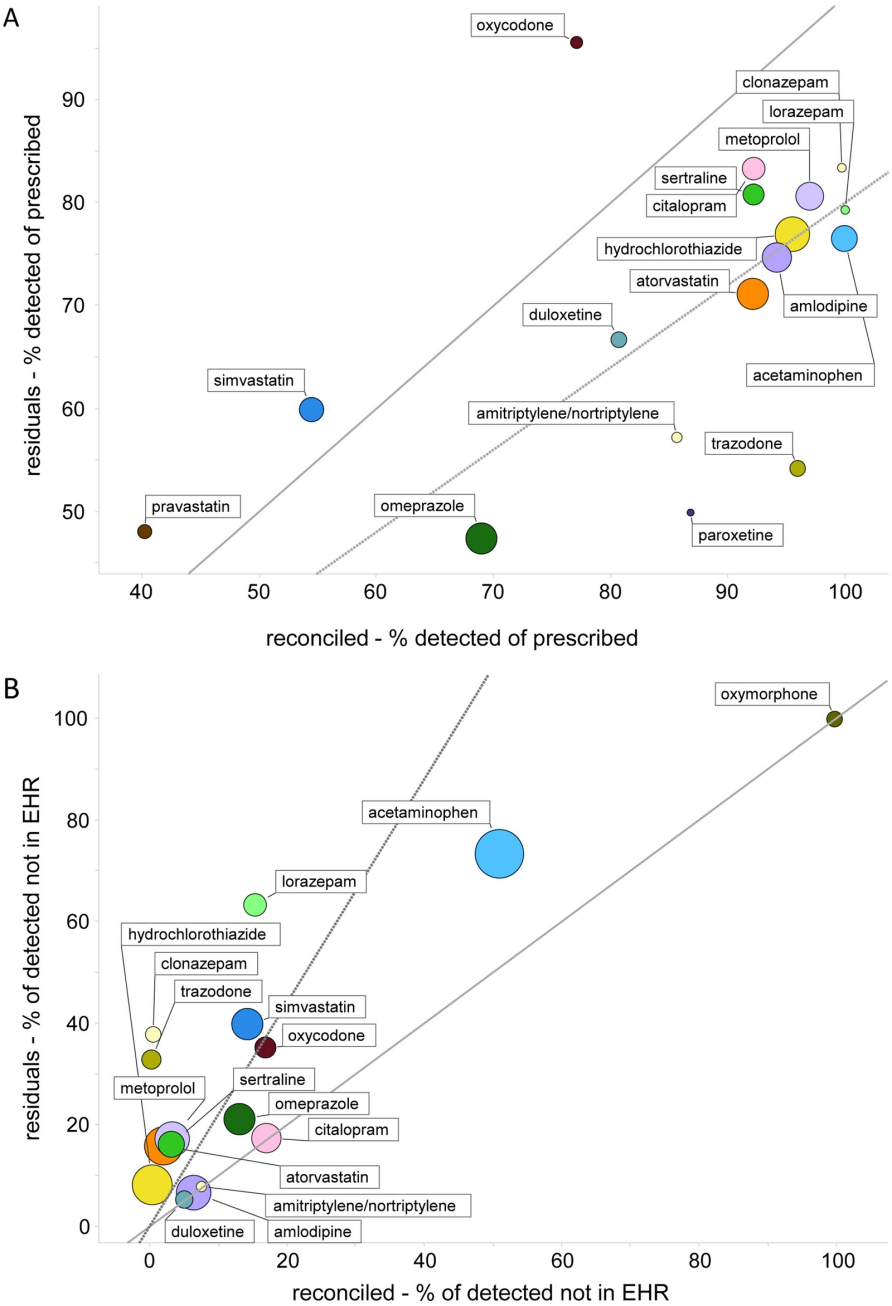
Adherence and non-prescribed medication use in two cohorts. A) Percent of prescribed medications that are detected (adherence), for medications having 10 or more prescriptions in each cohort. B) Percent of detected medications not in the EHR (non-prescribed), for medications having 10 or more detections in each cohort. The solid diagonal line indicates equality in both cohorts, and the dashed line indicates the overall ratio of adherence or non-prescribed use between cohorts, calculated across all medications. Markers are sized proportionally to log10 of prescriptions or detections.

We examined frequency trends for drugs that were detected in both cohorts. A higher proportion of prescribed metabolic agents, such as statin medications, were detected in the Residuals Cohort, while a larger proportion of prescribed antidepressants, including paroxetine and trazodone, were detected in the Reconciled Cohort (Fig 4A). Conversely, the proportion of detected medications not in the medical record was higher for over-the-counter analgesics such as ibuprofen and acetaminophen, and drugs of abuse, including benzodiazepines, in the Residuals Cohort than in Reconciled Cohort (Fig 4B).

**Fig 5:**
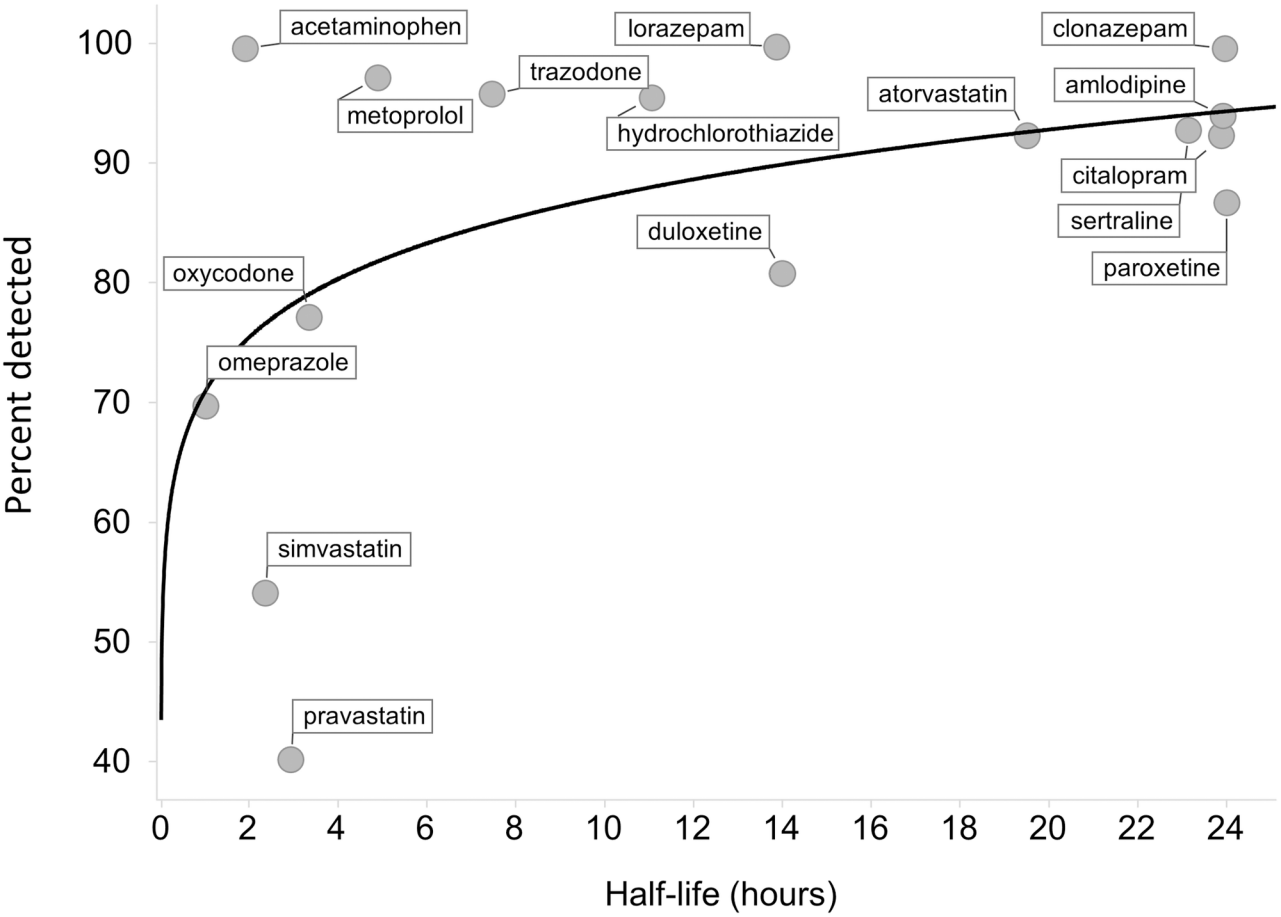
Percent of prescribed medications that are detected vs. medication half-life for Reconciled Cohort. Medications with half-life > 24 hours are shown at 24 hours on the abscissa. The fit denotes the least-squares power curve; the functional form was selected due to expected exponential decay of medication concentration with time.

Several drugs with lower levels of detection relative to prescribing rates have short oral half-lives, making them theoretically difficult to detect upon q.d. dosing. Therefore, we examined the proportion of detected medications as a function of drug half-life in the Reconciled Cohort, where we gathered self-reported time of dosing and where patients exhibited overall higher medication adherence (Fig 5). The percentage detected was generally lower for simvastatin, pravastatin and omeprazole, but not for acetaminophen and metoprolol. All these medications have average literature half-lives less than three hours (Table 2). Comparing simvastatin (t1/2 = 2.5 hours) and pravastatin (t1/2 = 2.9 hours) to atorvastatin (t1/2 = 20 hours) was instructive, as atorvastatin would be predicted to reach steady state blood concentrations upon q.d. dosing, whereas simvastatin and pravastatin would not based on oral half-life. The detection rate for atorvastatin (93%) exceeded the detection rates of the short-lived statins (55% simvastatin and 40% pravastatin). For drugs with half-lives less than four hours, we evaluated the percentage detected vs. time since last dose (S1 Fig). A decreasing trend of single point exposure vs. time since last dose for simvastatin was observed, but no such trend was observed with other short half-life medications, such as oxycodone. These empirical data show that many such drugs can be detected 12 hours or more after dosing.

**Fig 6:**
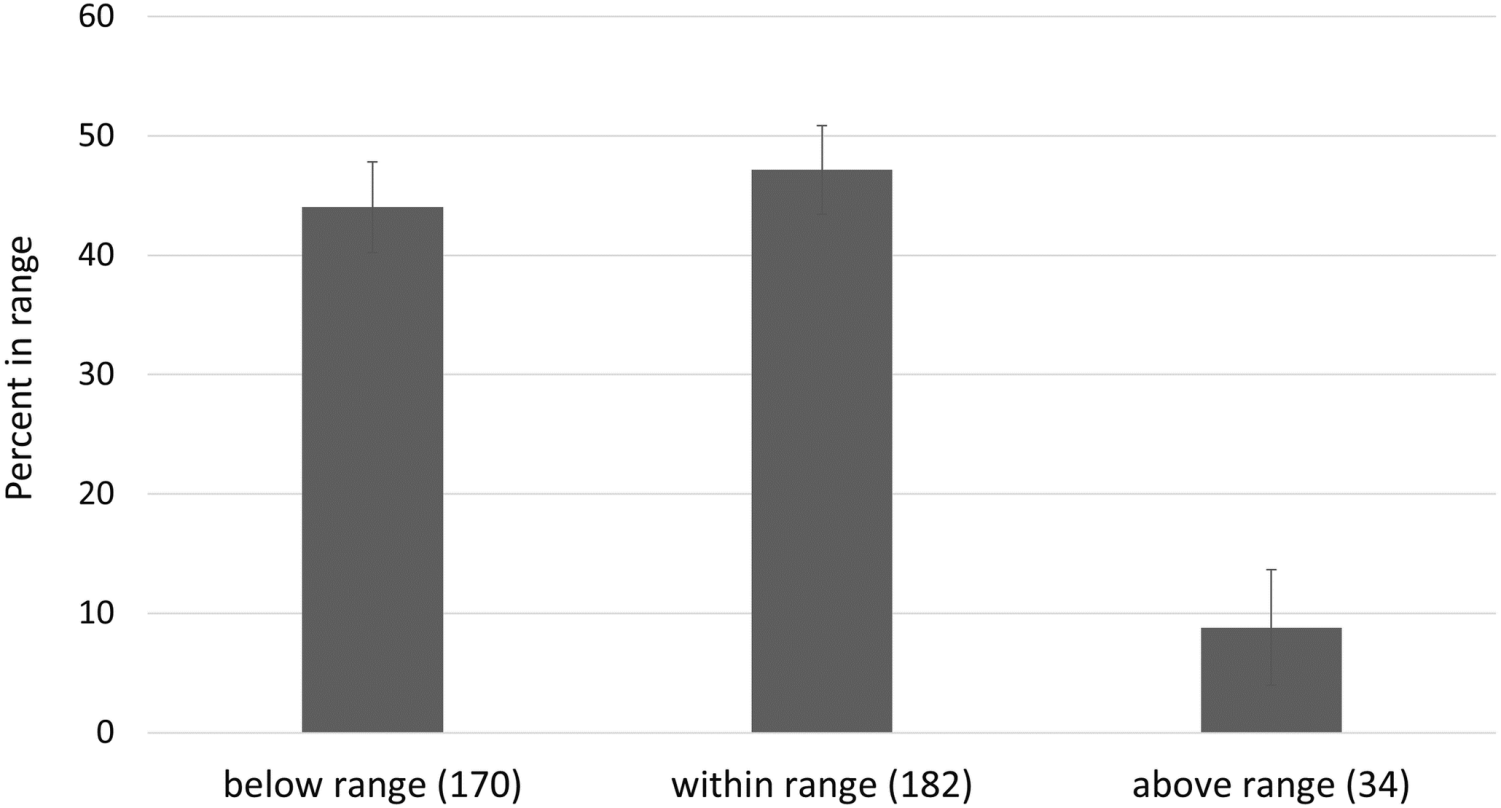
Medication detections vs. therapeutic monitoring ranges in Reconciled Cohort. Percent of medications detected quantitatively below, within or above ranges established in the therapeutic drug monitoring literature, for drugs that were listed in the patients EHR. Error bars were calculating from Bernoulli trials.

A central tenet in pharmacology is to optimize drug concentrations at the target to elicit the intended effect. In practice, measuring drug concentrations in blood is a useful surrogate for most medications, and the optimal blood levels have been established for many drugs. We compared the concentration of each medication detected to the published therapeutic reference range (Table 3; S2 Fig), focusing on the Reconciled Cohort, where patients took prescribed medications at a high rate. In this cohort 53% of detected drugs were observed to lie outside these ranges (Fig 6). Medications were more frequently detected at concentrations below the therapeutic reference range than at concentrations above the therapeutic reference range, and the percentage of drugs within, above, or below the therapeutic reference range was remarkably consistent between patient cohorts (Table 3). We explored the impact of dose and time since dose, and found modest predictive utility in explaining variation in drug levels (S2 Table)

**Table 3:** Prescribed and detected drug rates and levels vs. therapeutic drug range.

	Detected drugs per patient^a^		Percent of drugs by category^b^	
	Residuals Cohort	Reconciled Cohort	Residuals Cohort	Reconciled Cohort
DAP^c^	0.9	2.7	46	71
PND^c^	0.4	0.4	19	10
PND-prn^c^	0.2	0.2	11	6
DNP^c^	0.4	0.5	23	13
drugs below-range (all)^d^	0.4	1.1	48	44
drugs below-range (T1/2 < 4) ^d^	0.2	0.4	21	14
drugs in range^d^	0.4	1.2	45	47
drugs above range^d^	0.06	0.2	7	9

^a^ Number of drugs per patient in each category, and

^b^ percentage of drugs in each category.

^c^ Each prescribed and/or detected drug was assigned to one of 4 categories: detected and prescribed (DAP), prescribed not detected (PND), PND drugs taken as needed (PND-prn), and detected but not prescribed (DNP) drugs.

^d^ For detected and prescribed drugs that were measured quantitatively, tabulation by drug level compared to therapeutic drug ranges

## Discussion

We developed a 38-medication LC/MS/MS assay that crosses therapeutic indications for the detection and quantitation of medications in serum. We used the assay as a surrogate of medication adherence, a tool to improve medical record accuracy, and as a comprehensive method to measure exposure in patients. When reconciled with patient’s EHRs, medication measurement in serum offers an empirical measure of adherence and insight into EHR fidelity. Further, quantitative measurement in serum allowed for comparison of each detected medication concentration relative to the therapeutic reference range, elucidating the extent of patient exposure variability patients.

We queried the systemic circulation in two patient cohorts. The first, 821-patient cohort (Residuals Cohort) was designed to obtain samples from de-identified outpatients blinded to the medication testing paradigm. As such, comparisons between medications detected empirically and those in the prescription record would not be biased by patient behaviors associated with knowledge of drug testing. The second cohort of 151 patients (Reconciled Cohort) was prospectively enrolled, had medical records reconciled in a self-reporting interview, and consented to have blood tested for the presence of medications. In this cohort, we queried patients with reconciled records and a propensity to adhere to complex medication paradigms on how often medications would fall within the desired therapeutic reference range. Overall medication usage and detection rates were higher in this cohort and fewer medications were detected that were not listed in the medical record (Figs 1–3), producing the desired cohort to investigate quantitative aspects of medication exposure in complex patients that take medications as indicated.

In the residuals cohort, we found that 71% of prescribed drugs were detected in patients, a result slightly higher than estimates of compliance using pill counting and other methods of adherence measurement [37, 38]. Blood concentrations from most medications remain at detectable levels for several days post-ingestion, therefore slightly higher ‘adherence’ rates using medication monitoring relative to indirect methods likely results from patients that are partially adherent. The most frequently detected medications were drugs prescribed for metabolic and cardiovascular disease. The most disproportionately detected medications were of the psychotropic class, as enrollment criteria for the Reconciled Cohort required one psychotropic medication in the patient record prior to enrollment (Fig 2). Acetaminophen was the most often detected medication in circulation (Table 2). The frequency of detection and cumulative dose of this drug can become unintentionally high in patients, as this medication is found in at least 650 over-the-counter products, many of which are over-the-counter combination products taken simultaneously.

The rate of detection for medications that were not in the prescription record, the converse of the adherence measure discussed above, is novel information for the healthcare provider. Overall, 33% of detected medications in the Residuals Cohort were not in the medical record, with higher rates for over-the-counter medications, such as ibuprofen, and abused medications, such as benzodiazepines (Fig 4). This proportion decreased to 15% in the Reconciled Cohort, demonstrating that adherence and medical record omissions go hand in hand for the polypharmacy patient. Detected medications not in the EHR also create treatment issues, as drug-drug interactions with current treatment or future prescribing cannot be addressed when the medications are unbeknownst to the physician. This offers the opportunity for improving the medication reconciliation process and patient literacy [39–42].

The Reconciled Cohort was used to assess the impact of polypharmacy and biological factors on medication blood levels by testing in patients adherent to complex pharmacy regimens. Reference ranges were derived from published values for each medication in the assay panel, some of which had more supporting literature than others. Serum concentrations below the therapeutic reference range lower limit are unlikely to elicit a therapeutic response and concentrations above the upper limit exhibit tolerability decreases or no evidence that therapeutic improvement will be enhanced. This range is meant to be an orienting value, and is not necessarily applicable to all patients for each individual medication (26). More than half of the medications detected in this cohort were not within the therapeutic range (Fig 6). This finding deserves further study, including investigation into caveats associated with this type of measurement. Therapeutic drug monitoring has been performed with antipsychotic medications as single-medication studies in a variety of healthcare settings, and it has been consistently observed that medications are often out of range [26, 43, 44]. We now extend these findings to non-psychotropic medications, including medications more frequently prescribed to US patients alone or in combination [36]. Typically, therapeutic drug monitoring studies are conducted with patient medications at steady state and samples taken at trough levels. Although we did not replicate true trough sample collection with the present study design, given the multiplicity of medications tested at one time, our data demonstrate that almost 50% of medications are below the intended therapeutic reference range. This suggests that a significant number of patients have sub-therapeutic levels of medication when multiple medications prescribed.

We collected self-reported time of dosing in Reconciled Cohort patients. Although individual medication half-lives are expected to be important criteria when monitoring medication levels, the correlation of medication exposure with time of dosing varied widely (S1 Fig). The percentage of prescribed medications detected in patients was generally lower for simvastatin, pravastatin and omeprazole, but not for acetaminophen and oxycodone (Fig 5); these drugs have average literature oral half-lives less than 3 hours (Table 2). Reasons patients may be below the reference range are multifactorial and include; 1) patients may be partially adherent, with medication persistence lacking, 2) the therapeutic range, which is often developed in clinical trial patients lacking real-world diversity, may be inaccurate, or 3) pharmacokinetic drug-drug or drug-gene interactions may be manifesting in these polypharmacy patients. There are countless other reasons, including patient health and biological makeup, but the finding of extensive variability in medication exposure is important for optimizing medication therapy. As data accumulate with each medication measured, we will begin to address these issues by comparing measured data to patient outcomes, and de-convolute behavioral vs. biological factors underlying variability in drug treatment and response.

The current study included 38 medications, offering a comprehensive survey of the most frequently prescribed psychotropic medications and select over-the-counter and non-psychotropic medications used to treat other chronic diseases. In theory, the approach applied herein could be scaled to detect several hundred cross-therapeutic medications simultaneously, detecting a very high percentage of written prescriptions. Measuring the majority of frequently taken medications provides the healthcare professional a comprehensive view of therapy for the complex patient that cannot be obtained without empirical measurement, although one must consider the pharmacokinetic limitations that may hamper the detection or quantitation of a particular drug, such as topical administration or short half-life.

There are several limitations in the current study. First, the use of exposure as a surrogate of medication adherence, medical record accuracy, and therapeutic range has caveats given the current state of real-world medication exposure knowledge. Except for medications that are frequently monitored, such as digoxin or phenytoin, published information is lacking information on medication exposure relative to outcomes. For some medications, there have yet to be published studies linking blood levels to outcomes, and in a few, no association was shown to exist when assessed. The measurement of medications using the LC/MS/MS methodology deployed herein is highly precise and accurate, but there are a multitude of reasons a medication prescribed may not be detected. Finally, medication persistence, drug interactions, genetics, disease state, and many other factors contribute to whether a medication detected falls within published therapeutic reference ranges, and with errors in self-reported medication ingestion and therapeutic range derivation issues, it would be premature to use this information quantitatively as stand-alone decision criteria in medication management as it stands today. The best way to circumvent these issues is to collect real-world exposure information on more medications relative to patient outcomes, and build empirical measurement data into largely theoretical clinical decision support on medication exposure relative to response.

## Conclusions

These studies demonstrate using a novel and empirical surrogate approach that patients do not take all their prescribed medications, that the medication lists in EHRs are often erroneous, and that medication exposure is more variable than previously demonstrated. In these studies, only 37% of prescribed or ingested medications were fully in line with the medical record that the healthcare provider was working from. Ours is the first study to empirically measure cross therapy medication levels regardless of prescription record, and illustrates the scope of multifactorial problem underlying medication therapy management. We have shown with 38 medications that the issue of adherence and medical record accuracy is substantial, and expanding these studies to more complex patients, measuring more simultaneous medications, and gathering requisite genetic, wellness, and outcome data will prove valuable in explaining sources of medication exposure and its relevance to treating disease. The quantitative aspect of blood-based medication measurement deserves further study, and with increased sample size driving model building, can ultimately extend this approach beyond simple adherence and record reconciliation into exposure-based prescribing.

## Supporting information

S1 Fig Percent of prescribed medications that are detected vs. time since ingestion. Percent of prescribed medications that are detected for a given range of hours since taking (x-axis), using patient-reported medication ingestion times from Reconciled Cohort. Values on bars denote number of observations in the given time range. The absence of a count label indicates that there are no observations in that time range.
(TIF)

S2 Fig Distribution of log10 (concentration) aggregated across both cohorts. Vertical reference lines denote the low/high therapeutic drug range according to the literature. Value below the drug name denote its half-life in hours. Only drugs with 10 or more detections are shown.
(TIF)

S1 Table Reference ranges and assay performance for the medication panel. (DOCX)

S2 Table Relationship between drug concentration and patient-reported dose and time since taking medication in cohort 2. ^a^ Only drugs detected and prescribed 10 or more times; ^b^ patient-reported doses for detected drugs; ^c^ Spearman rho correlation between concentration vs. dose or time since dosing; ^d^ hydrochorothiazide.
(DOCX)

S1 Dataset De-identified patients, gender, age and summary of prescribed and detected drugs. (XLSX)

S2 Dataset Prescribed and/or detected drugs for two patient cohorts. (XLSX)

## References

[1] Aitken M, Kleinrock M. Global Use of Medicines 2020. Outlook and Implications. IMS Institute for Healthcare Informatics, 2015 November 2015. Report No.

[2] Kesselheim AS, Avorn J, Sarpatwari A. The High Cost of Prescription Drugs in the United States: Origins and Prospects for Reform. JAMA. 2016;316(8):858–71. doi: 10.1001/jama.2016.11237 27552619 10.1001/jama.2016.11237

[3] Dieleman JL, Baral R, Birger M, Bui AL, Bulchis A, Chapin A, et al US Spending on Personal Health Care and Public Health, 1996–2013. JAMA. 2016;316(24):2627–46. doi: 10.1001/jama.2016.16885 28027366 10.1001/jama.2016.16885 PMC5551483

[4] Horn S, Gassaway J. Practice-Based Evidence Study Design for Comparative Effectiveness Research. Med Care. 2007;45:S50–S7. doi: 10.1097/MLR.0b013e318070c07b 17909384 10.1097/MLR.0b013e318070c07b

[5] Albert NM. Improving Medication Adherence in Chronic Cardiovascular Disease. Critical Care Nurse. 2008;28(5):54–64. 18827087

[6] Choudhry NK, Krumme AA, Ercole PM, Girdish C, Tong AY, Khan NF, et al Effect of Reminder Devices on Medication Adherence: The REMIND Randomized Clinical Trial. JAMA Intern Med. 2017 doi: 10.1001/jamainternmed.2016.9627 28241271 10.1001/jamainternmed.2016.9627 PMC5470369

[7] Hefner G, Laib AK, Sigurdsson H, Hohner M, Hiemke C. The value of drug and metabolite concentration in blood as a biomarker of psychopharmacological therapy. Int Rev Psychiatry. 2013;25(5):494–508. doi: 10.3109/09540261.2013.836475 24151798 10.3109/09540261.2013.836475

[8] Czobor P, Skolnick P. The Secrets of a Successful Clinical Trial: Compliance, Compliance, and Compliance. Molecular Interventions. 4 2011;11(2):107–10. doi: 10.1124/mi.11.2.8 21540470 10.1124/mi.11.2.8 PMC3109858

[9] Traylor AH, Schmittdiel JA, Uratsu CS, Mangione CM, Subramanian U. Adherence to Cardiovascular Disease Medications: Does Patient-Provider Race/Ethnicity and Language Concordance Matter? J Gen Intern Med. 2010;25(11):1172–7. doi: 10.1007/s11606-010-1424-8 20571929 10.1007/s11606-010-1424-8 PMC2947630

[10] Cutler DM, Everett W. Thinking Outside the Pillbox—Medication Adherence as a Priority for Health Care Reform. The New England Journal of Medicine. 4 29, 2010;362(17):1553–5. doi: 10.1056/NEJMp1002305 20375400 10.1056/NEJMp1002305

[11] Mixon AS, Neal E, Bell S, Powers JS, Kripalani S. Care transitions: a leverage point for safe and effective medication use in older adults—a mini-review. Gerontology. 2015;61(1):32–40. doi: 10.1159/000363765 25277280 10.1159/000363765 PMC4479140

[12] Aitken M, Valkova S. Avoidable Costs in U.S. Healthcare. The $200 Billioin Opportunity from Using Medicines More Responsibly. IMS Institute for Healthcare Informatics, 2013 June. Report No.

[13] Kymes SM, Pierce RL, Girdish C, Matlin OS, Brennan T, Shrank WH. Association Among Change in Medical Costs, Level of Comorbidity, and Change in Adherence Behavior. Am J Manag Care. 2016;22(8):e295–e301. 27556832

[14] Kessler C, Ward MJ, McNaughton C. Reducing Adverse Drug Events. The Need to Rethink Outpatient Prescribing. JAMA. 2016;316(20):2092–3. doi: 10.1001/jama.2016.16392 27893112 10.1001/jama.2016.16392 PMC5779096

[15] Budnitz DS, Lovegrove MC, Shehab N, Richards C. Emergency Hospitalizations for Adverse Drug Events in Older Americans. The New England Journal of Medicine. 2011;365:2002–12. doi: 10.1056/NEJMsa1103053 22111719 10.1056/NEJMsa1103053

[16] Brummel A, Lustig A, Westrich K, Evans MA, Plank GS, Penso J, et al Best Practices: Improving Patient Outcomes and Costs in an ACO Through Comprehensive Medication Therapy Management. J Manag Care Pharm. 2014;20(12):1152–8. 25491911

[17] Costedoat-Chalumeau N, Pouchot J, Guettrot-Imbert G, Le Guern V, Leroux G, Marra D, et al Adherence to treatment in systemic lupus erythematosus patients. Best Pract Res Clin Rheumatol. 2013;27(3):329–40. doi: 10.1016/j.berh.2013.07.001 24238690 10.1016/j.berh.2013.07.001

[18] Osterberg L, Blaschke T. Adherence to Medication. N Engl J Med. 2005;353(5):487–97. doi: 10.1056/NEJMra050100 16079372 10.1056/NEJMra050100

[19] Gleason KM, McDaniel MR, Feinglass J, Baker DW, Lindquist L, Liss D, et al Results of the Medications at Transitions and Clinical Handoffs (MATCH) study: an analysis of medication reconciliation errors and risk factors at hospital admission. J Gen Intern Med. 2010;25(5):441–7. doi: 10.1007/s11606-010-1256-6 20180158 10.1007/s11606-010-1256-6 PMC2855002

[20] Mekonnen AB, McLachlan AJ, Brien JA. Effectiveness of pharmacist-led medication reconciliation programmes on clinical outcomes at hospital transitions: a systematic review and meta-analysis. BMJ Open. 2016;6(2):e010003 doi: 10.1136/bmjopen-2015-010003 26908524 10.1136/bmjopen-2015-010003 PMC4769405

[21] Phansalkar S, Her QL, Tucker AD, Filiz E, Schnipper J, Getty G, et al Impact of incorporating pharmacy claims data into electronic medication reconciliation. Am J Health Syst Pharm. 2015;72(3):212–7. doi: 10.2146/ajhp140082 25596605 10.2146/ajhp140082

[22] Kootstra-Ros JE, Van Weelden MJ, Hinrichs JW, De Smet PA, van der Weide J. Therapeutic drug monitoring of antidepressants and cytochrome p450 genotyping in general practice. J Clin Pharmacol. 2006;46(11):1320–7. doi: 10.1177/0091270006293754 17050797 10.1177/0091270006293754

[23] Crettol S, de Leon J, Hiemke C, Eap CB. Pharmacogenomics in psychiatry: from therapeutic drug monitoring to genomic medicine. Clin Pharmacol Ther. 2014;95(3):254–7. doi: 10.1038/clpt.2013.221 24196844 10.1038/clpt.2013.221

[24] Grundmann M, Kacirova I, Urinovska R. Therapeutic monitoring of psychoactive drugs—antidepressants: a review. Biomed Pap Med Fac Univ Palacky Olomouc Czech Repub. 2015;159(1):35–43. doi: 10.5507/bp.2013.020 23549513 10.5507/bp.2013.020

[25] Jin Y, Pollock BG, Frank E, Cassano GB, Rucci P, Muller DJ, et al Effect of age, weight, and CYP2C19 genotype on escitalopram exposure. J Clin Pharmacol. 2010;50(1):62–72. doi: 10.1177/0091270009337946 19841156 10.1177/0091270009337946 PMC3571021

[26] Hiemke C, Baumann P, Bergemann N, Conca A, Dietmaier O, Egberts K, et al AGNP Consensus Guidelines for Therapeutic Drug Monitoring in Psychiatry: Update 2011. Pharmacopsychiatry. 2011;44(6):195–235. doi: 10.1055/s-0031-1286287 21969060 10.1055/s-0031-1286287

[27] Dias E, Hachey B, McNaughton C, Nian H, Yu C, Straka B, et al An LC-MS assay for the screening of cardiovascular medications in human samples. J Chromatogr B Analyt Technol Biomed Life Sci. 2013;937:44–53. doi: 10.1016/j.jchromb.2013.08.010 24013190 10.1016/j.jchromb.2013.08.010 PMC3800555

[28] Cao Z, Kaleta E, Wang P. Simultaneous Quantitation of 78 Drugs and Metabolites in Urine with a Dilute-And-Shoot LC-MS-MS Assay. J Anal Toxicol. 2015;39(5):335–46. doi: 10.1093/jat/bkv024 25833899 10.1093/jat/bkv024

[29] Schultz M, Schmoldt A. Therapeutic and toxic blood concentrations of more than 500 drugs. Die Pharmazie [Internet]. 1997; 52(12):[895–911 pp.]. Available from: europepmc.org/abstract/med/9442555. 9442555

[30] Baer DM, Paulson RA, Haverstick DM. Cutoff and Toxicity Levels for Drugs-of-Abuse Testing. In: CLR20152016-Table-of-Cutoff-Toxicity-DOA.pdf, editor. 2015–2016.

[31] Schulz M, Iwersen-Bergmann S, Andresen H, Schmoldt A. Therapeutic and toxic blood concentrations of nearly 1,000 drugs and other xenobiotics. Crit Care. 2012;16(4):R136 doi: 10.1186/cc11441 22835221 10.1186/cc11441 PMC3580721

[32] Hammett-Stabler CA. Critical Values for Therapeutic Drug Levels. In: CLR20152016-Table-of-Critical-Values.pdf, editor. 2015–2016.

[33] Poggenborg RP, Videbaek L, Jacobsen IA. A Case of Amlodipine Overdose. Basic & Clinical Pharmacology and Toxicology. 2006;99:209–12. 16930293 10.1111/j.1742-7843.2006.pto_318.x

[34] DeGorter MK, Tirona RG, Schwarz UI, Choi YH, Dresser GK, Suskin N, et al Clinical and pharmacogenetic predictors of circulating atorvastatin and rosuvastatin concentrations in routine clinical care. Circ Cardiovasc Genet. 2013;6(4):400–8. doi: 10.1161/CIRCGENETICS.113.000099 23876492 10.1161/CIRCGENETICS.113.000099 PMC3922121

[35] Pan HY, DeVault AR, Swites BJ, Whigan D, Ivashkiv E, Willard DA, et al Pharmacokinetics and pharmacodynamics of pravastatin alone and with cholestyramine in hypercholesterolemia. Clin Pharmacol Ther. 1990;48(2):201–7. 2116260 10.1038/clpt.1990.136

[36] Sutherland JJ, Daly TM, Liu X, Goldstein K, Johnston JA, Ryan TP. Co-prescription trends in a large cohort of subjects predict substantial drug-drug interactions. PLoS One. 2015;10(3):e0118991 doi: 10.1371/journal.pone.0118991 25739022 10.1371/journal.pone.0118991 PMC4349653

[37] Bruckart J. Medication Adherence in America: A National Report Card. 2013:1–39.

[38] Viswanathan M, Golin CE, Jones CD, Ashok M, Blalock SJ, Wines RCM, et al Interventions to Improve Adherence to Self-administered Medications for Chronic Diseases in the United States. Ann Intern Med. 2012;157:785–95. doi: 10.7326/0003-4819-157-11-201212040-00538 22964778 10.7326/0003-4819-157-11-201212040-00538

[39] Bunting BA, Lee G, Knowles G, Lee C, Allen P. The hickory project: controlling healthcare costs and improving outcomes for diabetes using the asheville project model. Am Health Drug Benefits. 2011;4(6):343–50. 25126361 PMC4105733

[40] Bunting BA, Smith BH, Sutherland SE. The Asheville Project: clinical and economic outcomes of a community-based long-term medication therapy management program for hypertension and dyslipidemia. J Am Pharm Assoc (2003). 2008;48(1):23–31. doi: 10.1331/JAPhA.2008.07140 18192127 10.1331/JAPhA.2008.07140

[41] Cumbler E, Wald H, Kutner J. Lack of Patient Knowledge Regarding Hospital Medications. Journal of Hospital Medicine. 2010;5:83–6. doi: 10.1002/jhm.566 20013875 10.1002/jhm.566

[42] Roblek T, Vaupotic T, Mrhar A, Lainscak M. Drug-drug interaction software in clinical practice: a systematic review. Eur J Clin Pharmacol. 2015;71(2):131–42. doi: 10.1007/s00228-014-1786-7 25529225 10.1007/s00228-014-1786-7

[43] Schoretsanitis G, Stegmann B, Hiemke C, Grunder G, Schruers KR, Walther S, et al Pharmacokinetic patterns of risperidone-associated adverse drug reactions. Eur J Clin Pharmacol. 2016;72(9):1091–8. doi: 10.1007/s00228-016-2085-2 27376639 10.1007/s00228-016-2085-2

[44] Gjestad C, Westin AA, Skogvoll E, Spigset O. Effect of proton pump inhibitors on the serum concentrations of the selective serotonin reuptake inhibitors citalopram, escitalopram, and sertraline. Ther Drug Monit. 2015;37(1):90–7. doi: 10.1097/FTD.0000000000000101 24887634 10.1097/FTD.0000000000000101 PMC4297217

