# Supplementary figures and images for: Medication adherence, medical record accuracy, and medication exposure in real-world patients using comprehensive medication monitoring
Source: PLoS One. 2017 Sep 28;12(9):e0185471. doi: 10.1371/journal.pone.0185471 (PMC5619774; doi:10.1371/journal.pone.0185471)

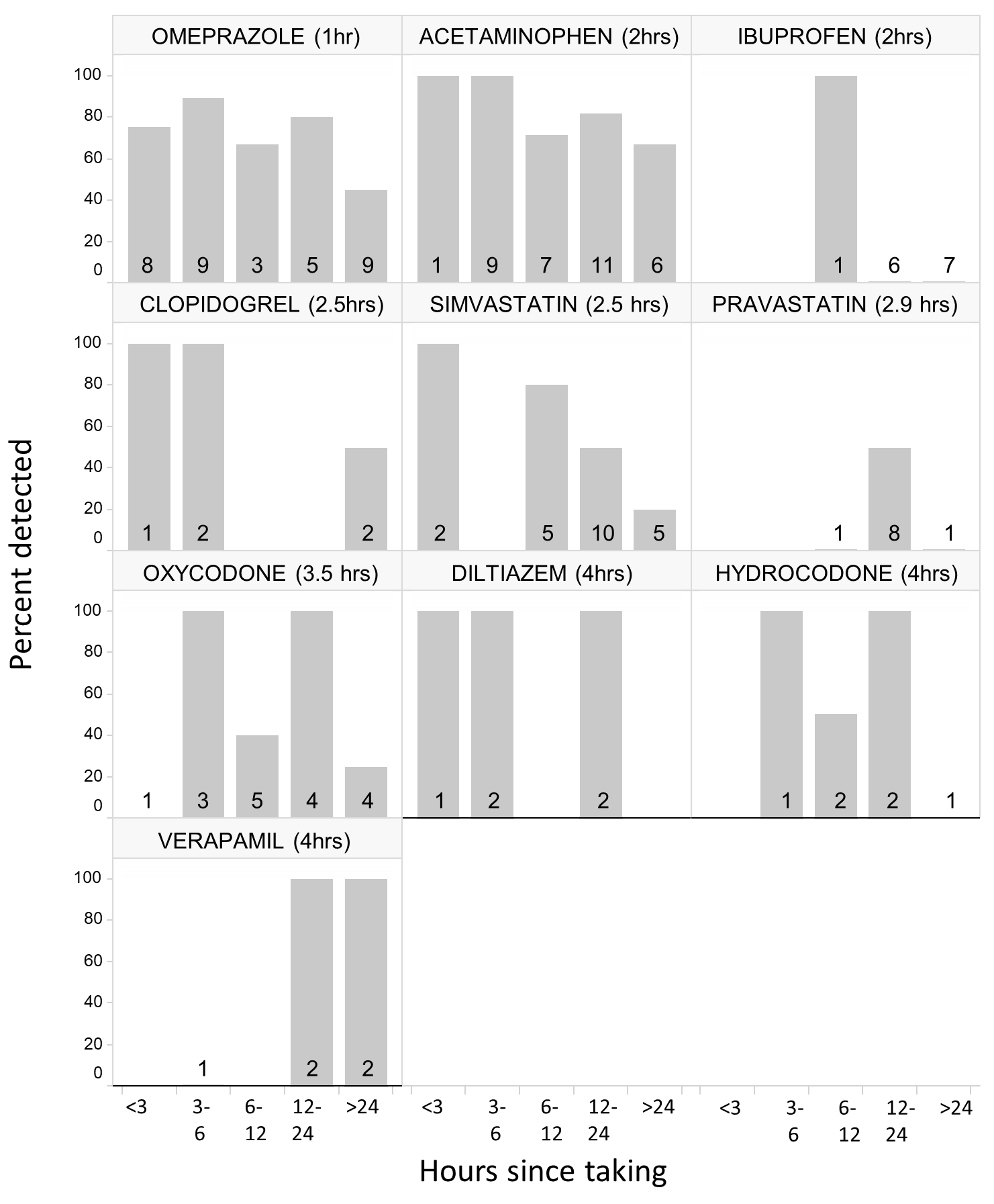

Supplement: S1 Fig — Percent of prescribed medications that are detected for a given range of hours since taking (x-axis), using patient-reported medication ingestion times from Reconciled Cohort. Values on bars denote number of observations in the given time range. The absence of a count label indicates that there are no observations in that time range. (TIF) [file pone.0185471.s001.tif]

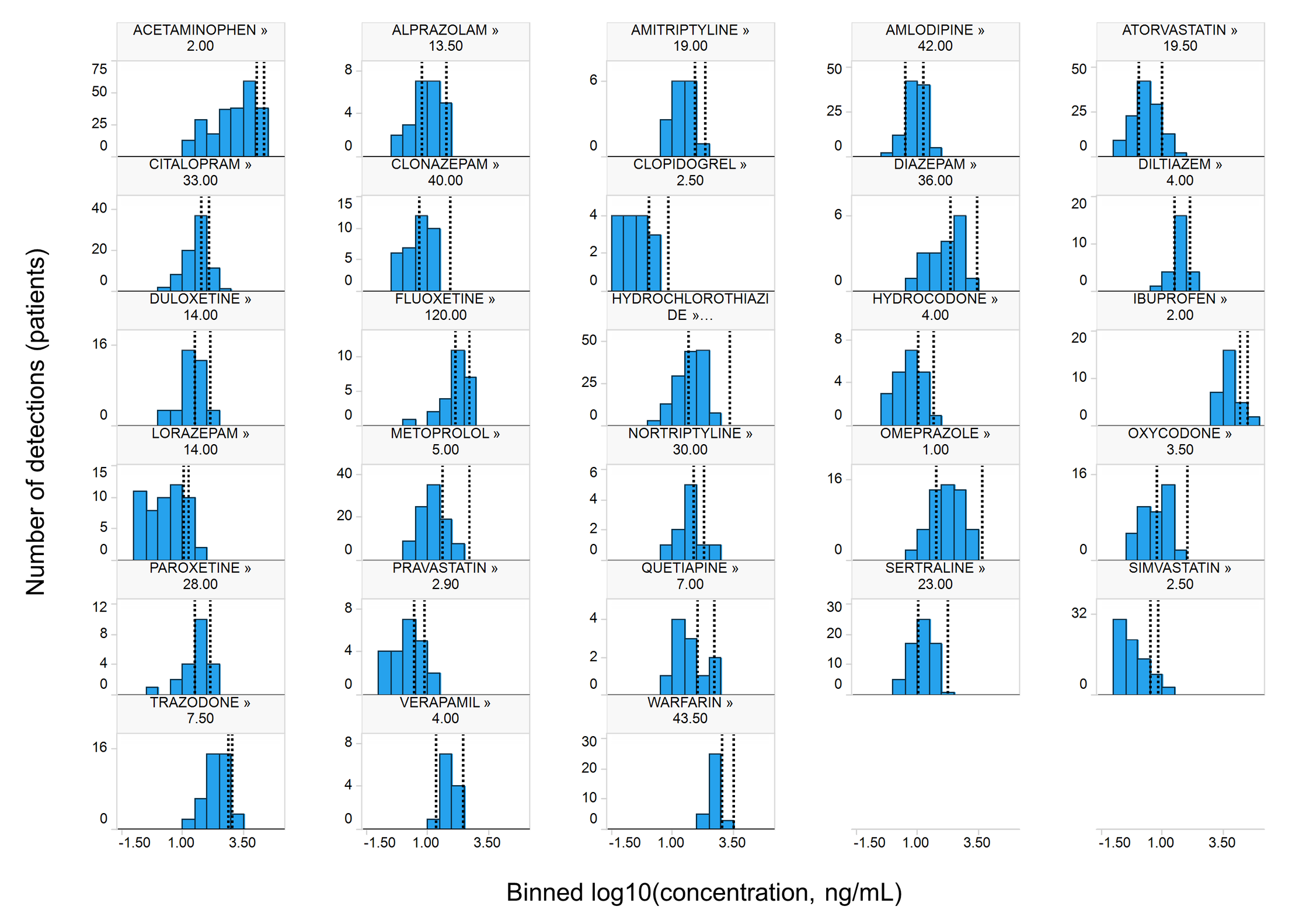

Supplement: S2 Fig — Vertical reference lines denote the low/high therapeutic drug range according to the literature. Value below the drug name denote its half-life in hours. Only drugs with 10 or more detections are shown. (TIF) [file pone.0185471.s002.tif]
