# Supplementary material for: Medication adherence, medical record accuracy, and medication exposure in real-world patients using comprehensive medication monitoring
Source: PLoS One. 2017 Sep 28;12(9):e0185471. doi: 10.1371/journal.pone.0185471 (PMC5619774; doi:10.1371/journal.pone.0185471)
Supplement: S1 Table — (DOCX) [file pone.0185471.s003.docx]

**S1 Table. Reference ranges and assay performance for the medication panel**

| **Medication** | **Low limit (ng/mL)** | **High limit (ng/mL)** | **Literature Reference** | **Inter-Assay Precision and Accuracy (*n*=74)** | | |
| --- | --- | --- | --- | --- | --- | --- |
|  |  |  |  | **QC Low Bias (%)** | **QC Low CV (%)** | **QC High Bias (%)** |
| acetaminophen | 10000 | 20000 | 31 | 14.0 | 32.6 | -1.2 |
| dihydrocodeine | 30 | 50 | 29 | 2.6 | 17.3 | -3.3 |
| hydrocodone | 10 | 40 | 30 | 3.0 | 15.3 | 0.2 |
| hydromorphone | 10 | 30 | 30 | 6.4 | 21.3 | 5.3 |
| ibuprofen | 15000 | 30000 | 31 | qual only |  |  |
| oxycodone | 5 | 100 | 30 | -1.6 | 12.8 | -1.8 |
| oxymorphone |  |  | NA | -6.4 | 14.5 | -1.4 |
| amitriptyline | 80 | 200 | 26 | -5.0 | 20.9 | -2.1 |
| citalopram | 50 | 110 | 26 | 1.7 | 13.4 | -0.4 |
| duloxetine | 30 | 120 | 31 | -4.7 | 20.0 | -5.2 |
| fluoxetine | 120 | 500 | 26 | -8.0 | 22.6 | -2.7 |
| nortriptyline | 70 | 170 | 26 | -3.6 | 23.3 | 1.8 |
| paroxetine | 30 | 120 | 26 | -4.6 | 18.8 | -4.4 |
| sertraline | 10 | 150 | 26 | -4.7 | 17.3 | -1.1 |
| trazodone | 700 | 1000 | 26 | -3.9 | 14.5 | -5.2 |
| clozapine | 350 | 600 | 26 | -1.3 | 11.7 | -1.6 |
| olanzapine | 20 | 80 | 26 | 1.8 | 12.6 | 0.0 |
| quetiapine | 100 | 500 | 26 | 1.6 | 15.8 | -0.9 |
| clonazepam | 4 | 80 | 31,26 | 2.5 | 13.0 | -0.3 |
| diazepam | 200 | 2500 | 31,26 | -7.0 | 13.8 | -8.8 |
| lorazepam | 10 | 15 | 31,26 | 0.7 | 16.2 | 0.3 |
| alprazolam | 5 | 50 | 31,26 | 1.6 | 18.6 | -2.3 |
| oxazepam | 200 | 1500 | 26 | -3.0 | 24.2 | -1.1 |
| temazepam | 20 | 900 | 26 | -5.0 | 15.3 | -2.1 |
| amiodarone | 500 | 2000 | 32 | -2.0 | 19.2 | -0.6 |
| amlodipine | 3 | 15 | 31,33 | -8.2 | 21.7 | -5.7 |
| atorvastatin | 1 | 9 | 34 | -1.6 | 11.3 | -0.5 |
| clopidogrel | 1 | 6 | 31 | -11.2 | 15.5 | -7.6 |
| diltiazem | 30 | 130 | 31 | -4.4 | 20.3 | -2.9 |
| gemfibrozil |  | 25 | 31 | qual only |  |  |
| hydrochlorothiazide | 40 | 2000 | 31 | -2.4 | 10.1 | -3.0 |
| metoprolol | 35 | 500 | 31 | -8.0 | 13.2 | -5.9 |
| pravastatin | 2.5 | 6.3 | 34,35 | -1.4 | 13.7 | 1.1 |
| simvastatin | 2.7 | 5.6 | 31 | -1.7 | 14.7 | -4.2 |
| verapamil | 20 | 250 | 31 | -1.4 | 15.4 | -4.1 |
| warfarin | 1000 | 3000 | 31 | -0.7 | 19.8 | -10.7 |
| omeprazole | 50 | 4000 | 31 | -2.2 | 18.7 | -8.8 |
| phenytoin | 10000 | 20000 | 26 | -2.6 | 22.1 | 1.7 |
