# Supplementary material for: Medication adherence, medical record accuracy, and medication exposure in real-world patients using comprehensive medication monitoring
Source: PLoS One. 2017 Sep 28;12(9):e0185471. doi: 10.1371/journal.pone.0185471 (PMC5619774; doi:10.1371/journal.pone.0185471)
Supplement: S2 Table — a Only drugs detected and prescribed 10 or more times; b patient-reported doses for detected drugs; c Spearman rho correlation between concentration vs. dose or time since dosing; d hydrochorothiazide. (DOCX) [file pone.0185471.s004.docx]

**S2 Table. Relationship between drug concentration and patient-reported dose and time since taking medication in cohort 2**

| Drug name^a^ | T_1/2_ (h) | N detect | N doses | Doses^b^ | ρ conc. vs dose^c^ | ρ conc. vs time^c^ |
| --- | --- | --- | --- | --- | --- | --- |
| OMEPRAZOLE | 1 | 15 | 2 | 20, 40 | 0.12 | -0.54 |
| ACETAMINOPHEN | 2 | 28 | 4 | 325, 500, 1000, 2000 | 0.01 | -0.53 |
| HCTZ^d^ | 11 | 45 | 3 | 12.50, 25, 50 | 0.24 | -0.70 |
| METOPROLOL | 5 | 29 | 6 | 12.5, 25, 50, 100, 125, 200 | 0.21 | -0.21 |
| DULOXETINE | 14 | 17 | 5 | 20, 30, 60, 90, 120 | 0.47 | -0.17 |
| LORAZEPAM | 14 | 11 | 3 | 0.25, 0.5, 1 | 0.02 | 0.05 |
| ATORVASTATIN | 19.5 | 36 | 6 | 5, 10, 20, 40, 60, 80 | 0.38 | -0.35 |
| SERTRALINE | 23 | 25 | 6 | 25, 50, 75, 100, 150, 200 | 0.42 | -0.09 |
| PAROXETINE | 28 | 13 | 7 | 10, 15, 20, 25, 30, 40, 60 | 0.84 | 0.07 |
| CITALOPRAM | 33 | 24 | 4 | 10, 15, 20, 40 | 0.69 | -0.08 |
| CLONAZEPAM | 40 | 11 | 3 | 0.5, 1, 2 | 0.04 | -0.67 |
| AMLODIPINE | 42 | 31 | 3 | 2.50, 5, 10 | 0.63 | -0.21 |

^a^ Only drugs detected and prescribed 10 or more times; ^b^ patient-reported doses for detected drugs; ^c^ Spearman rho correlation between concentration vs. dose or time since dosing; ^d^ hydrochorothiazide
